# Ectopic overexpression of the cell wall invertase gene *CIN1* leads to dehydration avoidance in tomato

**DOI:** 10.1093/jxb/eru448

**Published:** 2014-11-11

**Authors:** Alfonso Albacete, Elena Cantero-Navarro, Dominik K. Großkinsky, Cintia L. Arias, María Encarnación Balibrea, Roque Bru, Lena Fragner, Michel E. Ghanem, María de la Cruz González, Jose A. Hernández, Cristina Martínez-Andújar, Eric van der Graaff, Wolfram Weckwerth, Günther Zellnig, Francisco Pérez-Alfocea, Thomas Roitsch

**Affiliations:** ^1^Department of Plant Nutrition, CEBAS-CSIC,Campus de Espinardo, 30100 Murcia, Spain; ^2^Institute of Plant Sciences, Department of Plant Physiology, University of Graz, 8010 Graz, Austria; ^3^Department of Plant and Environmental Sciences, Copenhagen Plant Science Centre, University of Copenhagen,Højbakkegård Allé 13, DK-2630 Taastrup, Denmark; ^4^Centro de Estudios Fotosintéticos y Bioquímicos, Universidad Nacional de Rosario, Suipacha 531, 2000 Rosario, Argentina; ^5^Departamento de Agroquímica y Bioquímica, Facultad de Ciencias, Universidad de Alicante, 03080 Alicante, Spain; ^6^Department of Molecular Systems Biology, Faculty of Life Sciences, University of Vienna, 1090 Vienna, Austria; ^7^Instituto de Bioquímica Vegetal y Fotosíntesis, Universidad de Sevilla,CSIC, 41092 Sevilla, Spain; ^8^Department of Fruit Breeding, CEBAS-CSIC, Campus de Espinardo, 30100 Murcia, Spain; ^9^Global Change Research Centre, Czech Globe AS CR, v.v.i., Drásov 470, Cz-664 24 Drásov, Czech Republic

**Keywords:** Cell wall invertase, cytokinins, drought stress, ethylene, source–sink relationships, tomato.

## Abstract

Drought stress conditions modify source–sink relations, thereby influencing plant growth, adaptive responses, and consequently crop yield. Invertases are key metabolic enzymes regulating sink activity through the hydrolytic cleavage of sucrose into hexose monomers, thus playing a crucial role in plant growth and development. However, the physiological role of invertases during adaptation to abiotic stress conditions is not yet fully understood. Here it is shown that plant adaptation to drought stress can be markedly improved in tomato (*Solanum lycopersicum* L.) by overexpression of the cell wall invertase (cwInv) gene *CIN1* from *Chenopodium rubrum*. *CIN1* overexpression limited stomatal conductance under normal watering regimes, leading to reduced water consumption during the drought period, while photosynthetic activity was maintained. This caused a strong increase in water use efficiency (up to 50%), markedly improving water stress adaptation through an efficient physiological strategy of dehydration avoidance. Drought stress strongly reduced cwInv activity and induced its proteinaceous inhibitor in the leaves of the wild-type plants. However, the *CIN1*-overexpressing plants registered 3- to 6-fold higher cwInv activity in all analysed conditions. Surprisingly, the enhanced invertase activity did not result in increased hexose concentrations due to the activation of the metabolic carbohydrate fluxes, as reflected by the maintenance of the activity of key enzymes of primary metabolism and increased levels of sugar-phosphate intermediates under water deprivation. The induced sink metabolism in the leaves explained the maintenance of photosynthetic activity, delayed senescence, and increased source activity under drought stress. Moreover, *CIN1* plants also presented a better control of production of reactive oxygen species and sustained membrane protection. Those metabolic changes conferred by *CIN1* overexpression were accompanied by increases in the concentrations of the senescence-delaying hormone *trans*-zeatin and decreases in the senescence-inducing ethylene precursor 1-aminocyclopropane-1-carboxylic acid (ACC) in the leaves. Thus, cwInv critically functions at the integration point of metabolic, hormonal, and stress signals, providing a novel strategy to overcome drought-induced limitations to crop yield, without negatively affecting plant fitness under optimal growth conditions.

## Highlight

Overexpression of a cell wall invertase gene promotes dehydration avoidance and WUE, and delays senescence in tomato through limiting stomatal conductance and inducing sink metabolism and photosynthesis in source leaves.

## Introduction

The prospect of global warming coinciding with the continuous increase in the human population is projected to have a significant impact on the demands imposed on contemporary agricultural production. Abiotic stresses in general, and, more specifically, drought stress, have a strong negative impact on crop yield, because they create suboptimal growth conditions. Therefore, drought tolerance has attracted much attention and investment from private, public, academic, and philanthropic sectors (Marris, 2008; Lybbert and Bell, 2010). There is an urgent need to generate plants that are optimized in terms of tolerance to reduced water quantity and quality to support growth and development (Lybbert and Bell, 2010), which most probably cannot be achieved using only traditional methods of plant breeding. Drought stress leads to physiological modifications such as reduced photosynthesis, transcriptional and post-transcriptional regulation of various genes, and osmolyte biosynthesis (Seki *et al.*, 2007; Shinozaki and Yamaguchi-Shinozaki, 2007). Classic genetic engineering approaches involved target genes that function in mechanisms used by plants to avoid and/or tolerate drought, such as stomatal conductance, ionic homeostasis, or osmolyte production (Hirayama and Shinozaki, 2010), which, however, typically resulted in a trade-off causing reduced fitness or yield penalties under normal growth conditions. Such genes, frequently identified through expression profiling, include signalling components and downstream effector genes (Skirycz *et al.*, 2011). However, the advances provided by modern plant molecular biology and genetics could identify a new class of target genes (Webster *et al.*, 2012) linked to useful traits such as resistance to drought, salt, and other abiotic stress conditions, without negative impacts on plant fitness.

Plant fitness is intrinsically linked to the ability to produce and consume carbohydrates in a tissue-specific manner. In higher plants, growth and metabolism of sink tissues is sustained by the carbohydrates synthesized in source leaves, which are transported mainly as sucrose through the phloem into the sink tissues (Koch, 2004; Roitsch and González, 2004). Source–sink relationships are dynamic and change during development and in response to different biotic and abiotic stresses (Roitsch, 1999; Balibrea *et al*., 2000 2003; Roitsch and González, 2004; Albacete *et al.*, 2014*b*). In general, under abiotic stress conditions, the competition between different physiological processes and sink organs for the limited carbon supplies leads to a reduction in the sink strength, affecting overall plant growth and crop yield (Cuartero and Fernández-Muñoz, 1998). The use of sucrose in the sink tissues requires cleavage of the glycosidic bond, catalysed by both sucrose synthase and invertases. Three types of invertase isoenzymes are distinguished based on solubility, subcellular localization, pH optima, and isoelectric point: vacuolar invertase (vacInv), cytoplasmic invertase (cytInv), and cell wall-bound invertase (cwInv) (Roitsch and González, 2004). CwInv has been shown to play a crucial role in plant development by regulating sink strength, ensuring the steady supply of photoassimilates to sink tissues (Tang *et al.*, 1999; Goetz *et al.*, 2001; Roitsch *et al.*, 2003; Weschke *et al.*, 2003). Under water stress, differences in storage carbohydrate accumulation in drought-sensitive and drought-tolerant wheat were correlated with differences in sugar profiles, expression of cwInv genes, and levels of fructan biosynthesis in the anther and ovary (Ji *et al.*, 2010). Also, Koonjul *et al.* (2005) reported that transitory water deficit in wheat during male meiosis selectively down-regulated the transcription of two genes encoding a vacuolar (*Ivr5*) and a cell wall (*Ivr1*) invertase isoform in the anthers. It has been suggested that pollen sterility, or the concomitant inhibition of starch accumulation in water-stressed rice plants, is unlikely to be caused by carbohydrate starvation *per se*. Instead, an impairment of enzymes of sugar metabolism and starch synthesis may be among the potential causes of this failure (Sheoran and Saini, 1996). Nevertheless, the physiological mechanisms involved in altered assimilate partitioning and tolerance towards abiotic stress by invertases remain unclear (Albacete *et al.*, 2010 2011 2014*b*; Pérez-Alfocea *et al.*, 2010).

The increase of sink strength through cwInv activity is a general response under stress conditions. In particular, expression of the *CIN1* cwInv gene from *C. rubrum* has been reported to increase in response to a number of different stress-related stimuli (Roitsch and Ehness, 2000). In suspension-cultured cells, the inducing effect of the fungal elicitor chitosan could be mimicked by phosphatase inhibitors and benzoic acid (Ehness and Roitsch, 1997). Mechanical wounding of source leaves of *Chenopodium rubrum* plants also resulted in increased *CIN1* mRNA levels, showing the wide range of stress-related stimuli affecting *CIN1* expression (Roitsch *et al.*, 1995). In addition, cwInv is regulated at transcriptional and post-translational levels by many different factors including sugars (Roitsch *et al.*, 1995 2003; Roitsch, 1999), phytohormones (Roitsch *et al.*, 2003; Balibrea *et al.*, 2004; Roitsch and González, 2004), and proteinaceous inhibitors (Rausch and Greiner, 2004; Bonfig *et al.*, 2010; McKenzie *et al.*, 2013). Together, these mechanisms allow a fine-tuned regulation of the cwInv activity to control growth, development, and, therefore, plant adaptation under abiotic stress conditions. CwInv thus works as a pivotal enzyme at the integration point of metabolic, hormonal, and stress signals (Proels and Roitsch, 2009). In this study, it is shown that overexpression of the cwInv gene *CIN1* in tomato dramatically increases whole-plant water use efficiency (WUE) under water stress conditions, through a strategy of dehydration avoidance, providing a novel approach to overcome drought-induced limitations to crop productivity.

## Materials and methods

### *InvLp6g::CIN1* construction and overexpression

The full-length 1.7kb *CIN1* cDNA under the control of a 2.5kb fragment of the promoter of vacuolar invertase *pInvLp6g* from *Solanum pimpinellifolium* (Elliott *et al.*, 1993) (GenBank accession no. Z12028.1) was cloned into the vector pBI101. After transfer into the *Agrobacterium tumefaciens* strain LBA4404, cotyledons from the cv. P-73 of tomato (*Solanum lycopersicum* L.) were transformed with the *CIN1* overexpression construct. T_2_ plants from five different transgenic lines containing the *InvLp6g::CIN1* construct were identified as transgenic (homozygous or heterozygous) or azygous for the T-DNA based on the presence of the marker gene *NPTII* that confers resistance to the antibiotics kanamycin and neomycin determined by PCR. Subsequently, the homozygous or heterozygous transgenic state of the (PCR positive) T_2_ plants was determined in their respective T_3_ progeny by PCR.

### Plant growth conditions

Wild-type plants from the P-73 cultivar (WT) were used as controls. Tomato seeds were germinated at 28 °C and 90% relative humidity, and grown under 25/18 °C (day/night temperatures), 16h light (245 μmol m^–2^ s^–1^) and a relative humidity of 60–70%. At 15 d after sowing, 12 plants of each transgenic line and the WT were transferred to 10 litre pots filled with peat and grown for 14 d. At this point, watering was withheld for a period of 9 d. Three plants per line were irrigated at field capacity during this period and used as controls.

### *CIN1* expression analysis

Fresh tissues from mature leaves, roots, seedlings, and fruits were used for total RNA isolation, and 1 μg of total RNA was used for first-strand cDNA synthesis according to standard methods, using oligo(dT) primers. Semi-quantitative real-time PCR (RT-PCR) using actin to normalize the obtained cDNA amounts was performed as described previously (Großkinsky *et al.*, 2011). For *CIN1* expression analyses, the primers CIN1-Forward (5’-CCTGGGAGTATAGTGGCTGAACC-3’) and CIN1-Reverse (5’-AGGTCTTCTCTGAATCCG-3’) were used.

### Soil water potential and relative water content

Measurements of the soil water potential were done with a Watermark Soil Moisture Meter. Leaf relative water content (RWC) was determined as: RWC=(fresh weight–dry weight)/(turgid weight–dry weight). To determine the turgid weight, leaves were kept in distilled water in darkness at 4 ºC to minimize respiration losses until they reached a constant weight (full turgor, typically after 24h).

### Sucrolytic and other carbon metabolism enzyme assays, and invertase inhibitor activity

Sucrolytic and other carbon metabolism enzyme activities were assayed by determining the NADH delivered in a coupled enzymatic reaction using specific substrates/enzymes depending on the target enzyme (Balibrea *et al*., 1999 2003). The absorbance was monitored at 340nm. The proteins were analysed with Bradford reagent using bovine serum albumin (BSA) as standard. The invertase inhibitor assay was performed as previously described (Bonfig *et al.*, 2010).

### Sugar determination

A 100mg aliquot of leaf plant material was ground in liquid nitrogen and 0.9ml of water was added. After homogenization with cationic and anionic exchange resins and centrifugation for 10min at 20 000 *g* and 4 ºC, the supernatant was filtered and 10 μl were injected in a normal-phase liquid chromatography system (Shimadzu Corporation, Kyoto, Japan), using acetonitrile/water (85/15, v/v) as the mobile phase at a flow rate of 1ml min^–1^.

### CO_2_ exchange measurements

Gas exchange measurements were conducted in the fifth fully expanded leaf in each genotype with a gas exchange system (LI-6400; Li-Cor, Lincoln, NE, USA). Leaves were first equilibrated at a photon density flux of 500 μmol m^–2^ s^–1^ for at least 2min. After this, photosynthesis was induced with a photon density flux of 1000 μmol m^–2^ s^–1^ and 400 μmol mol^–1^ CO_2_ surrounding the leaf (*C*_a_). Leaf temperature was maintained at 25 ºC, and the leaf to air vapour pressure deficit was kept between 1 kPa and 1.3 kPa for the determination of the photosynthetic rate (*A*).

### Transmission electron microscopy

For ultrastructural studies, small pieces of mature leaves were fixed in 2.5% glutardialdehyde/2% paraformaldehyde in 0.06M cacodylate buffer (pH 7.2) for 90min, post-fixed, dehydrated, and embedded as previously described (Zechmann *et al.*, 2007). Ultrathin sections (80nm) were investigated after post-staining with uranyl aceate and lead citrate with a Philips CM10 transmission electron microscope.

### Accumulated transpiration and water use efficiency

Transpired water was measured gravimetrically by daily weighing the potted plants during the experiment. A pot with the same amount of soil but without a plant was weighed daily and used as a reference to determine the amount of water evaporated. WUE was determined as the biomass generated during the drought period (in grams) divided by the accumulated transpiration during that period (in millilitres).

### Chlorophyll fluorescence

Modulated chlorophyll fluorescence was measured in tagged and dark-adapted (30min) leaves, using a chlorophyll fluorometer OS-30 (OptiSciences, Herts, UK) with an excitation source intensity of 3000 μmol m^–2^ s^–1^. A special version of an Imaging-PAM Chlorophyll Fluorometer (Walz) was used to investigate spatio-temporal changes in photosynthetic parameters (Schreiber, 2004).

### Antioxidant enzymes

The leaf apoplastic fraction was isolated by vacuum infiltration in the presence of 50mM TRIS-acetate buffer pH 6.0. Samples were concentrated and pre-purified by chromatography on Sephadex G-25 NAP-10 columns (GE Healthcare) (Hernández *et al.*, 2001). Leaf residues (2g), which resulted from the apoplastic extraction, were homogenized using a mortar and pestle in 4ml of ice-cold 50mM TRIS-acetate buffer pH 6.0 containing 0.1mM EDTA, 2mM cysteine, and 0.2% (v/v) Triton X-100, and used as the symplastic fraction. Peroxidase (POX) and superoxide dismutase (SOD) activities were assayed as described previously (Hernández *et al.*, 2001).

### Glutathione and electrolyte leakage determination

Total and oxidized glutathione were extracted and analysed by high-performance liquid chromatography (HPLC) as described previously (Kranner and Grill, 1995). For electrolyte leakage measurements, tomato leaves (2g) were cut into pieces (~2cm^2^) and incubated in 8ml of MilliQ water in sealed tubes, for 2h at room temperature. After incubation, the conductivity of the bathing solution was measured with a conductivity meter. This value was referred to as value A. The bathing solutions were returned to the sealed tubes, containing the pieces of leaves, which were then incubated in a water bath at 95 ºC for 25min. After cooling to room temperature, the conductivity of the bathing solution was measured again. This is referred to as value B. For each measurement, electrolyte leakage was expressed as percentage leakage: [(value A/value B)×100].

### Proteomic analysis

Two-dimensional DIGE minimal labelling (Alban *et al.*, 2003) was used to identify leaf apoplast protein abundance differences. Gel image analysis was performed using Progenesis SameSpots v3.0 (Nonlinear Dynamics, Newcastle, UK) as described previously (Martínez-Esteso *et al.*, 2011). Spots whose normalized volume (% total spot volume) increased or decreased according to the treatment across the experiment were selected based on analysis of variance (ANOVA; *P*<0.05). Selected spots were manually excised from the gels and processed for identification by matrix-assisted laser desorption/ionization-time of flight (MALDI-TOF) and liquid chromatography coupled to tandem mass spectrometry (LC-MS/MS) in the PROTEORED^©^ proteomic facility at the University of Alicante (Spain). Protein in-gel digestion and identification by MALDI-TOF or LC-MS/MS and database search were done as described previously (Martínez-Esteso *et al.*, 2009).

### Metabolite profiling

Metabolite profiling was performed using gas chromatography coupled to a LECO Pegasus IV time-of-flight (Leco Corp Inc., St. Joseph, MI, USA) mass analyser (GC-TOF-MS) as previously described (Scherling *et al.*, 2009).

### Hormone extraction and analysis

Hormones were analysed as described previously (Albacete *et al.*, 2008).

### Statistics

All experiments were repeated three times, and the results of one representative experiment are presented in each case. Data were subjected to an ANOVA using the SPSS software (Version 19.0, SPSS Inc., Chicago, IL, USA). The statistical significance of the results was analysed by Student–Newman–Keuls test at the 5% level.

## Results

### *CIN1* overexpression increases water use efficiency and photosynthesis under drought stress

**Fig. 1.**
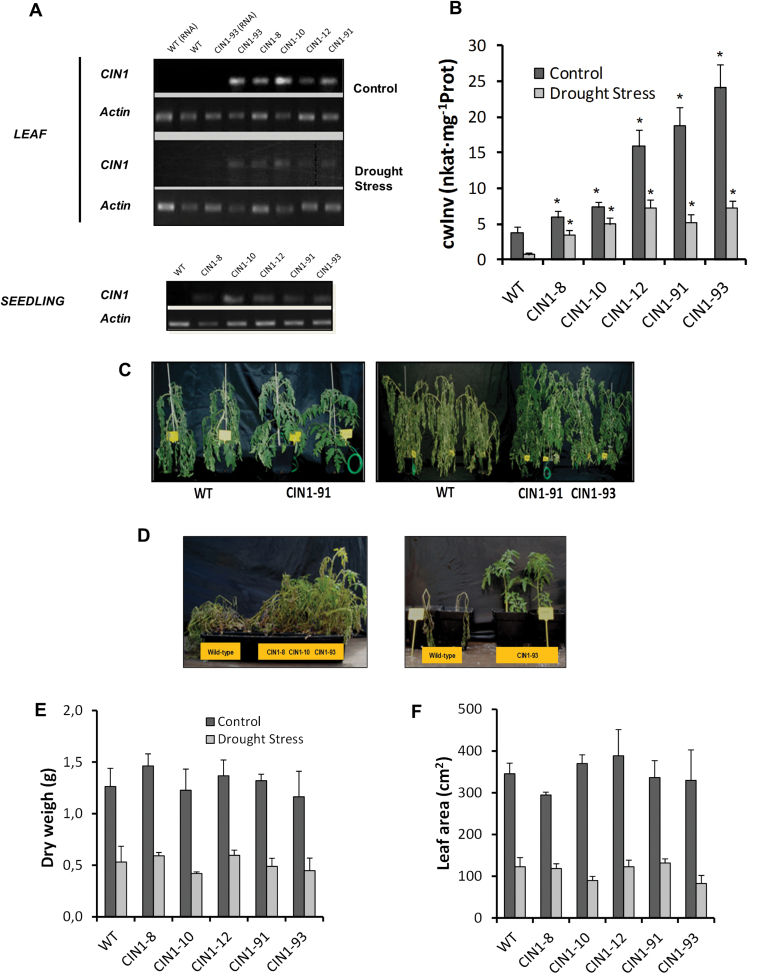
Expression and regulation of the *CIN1* gene (A) and cell wall invertase activity (B) in mature leaves of WT and *CIN1* plants under normal watering regimes and after 9 d of drought stress. Comparative images of WT and *CIN1* lines at the vegetative (7 d of drought stress, left panel) and flowering (2 d of drought stress, right panel) stages (C), and between WT and *CIN1* seedlings growing in a greenhouse under non-controlled conditions after 15 d of withholding water (left panel) and re-watering for 1 d (right panel) (D). Dry weight (E) and leaf area (F) of WT and *CIN1* plants under normal watering regimes and after 9 d of drought stress. Data are presented as means± SE, *P<0.05, one-way ANOVA, *n*=3.

It has recently been shown that transgenic tomato plants overexpressing the cwInv gene *CIN1* from *C. rubrum* under the control of the putative fruit-specific promoter *InvLp6g* recover sink strength and fruit growth under suboptimal conditions imposed by salinity (Albacete *et al.*, 2014*a*). Although the *InvLp6g* promoter should predominantly confer expression in tomato fruits, semi-quantitative RT-PCR analyses for five selected independent homozygous T_3_ lines revealed that the *CIN1* transgene is also expressed in seedlings and in leaves from plants grown under control conditions (Fig. 1A). Therefore, the effect of *CIN1* overexpression on whole-plant physiology was also investigated to distinguish between fruit-specific and systemic, general changes in physiology. Furthermore, overexpression of *CIN1* dramatically increased cwInv activity in the leaves of transgenic lines compared with the WT under normal watering regimes (Fig. 1B). Therefore, invertase activity was used as a more direct and specific indicator of the ‘performance’ of the transgenics rather than *CIN1* expression. Consequently, three groups of plant lines could be considered: (i) lines *CIN1-*12, *CIN1-*91, and *CIN1-*93 which all behave similarly to high cwInv activity lines; (ii) line *CIN1-*10 as an intermediate cwInv activity line; and (iii) line *CIN1-*8 as a low activity line, similar to the WT despite showing *CIN1* expression.

**Fig. 2.**
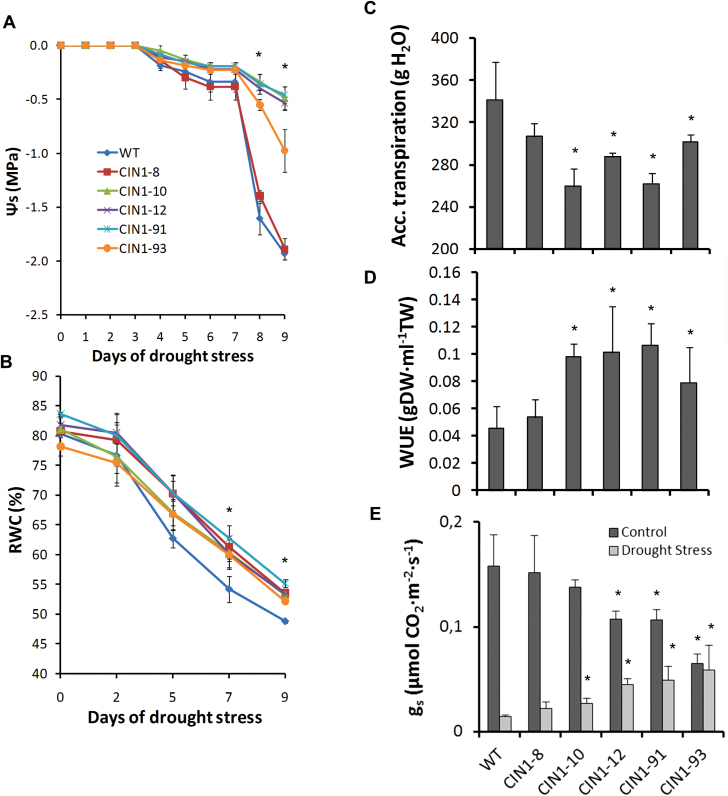
Soil water potential (A) and leaf relative water content (B) of WT and *CIN1* plants during the drought period. Accumulated transpiration (C), water-use efficiency (D), and stomatal conductance (E) in WT and *CIN1* plants after 9 d of drought stress. Data are presented as means ±SE., *P<0.05, one-way ANOVA, *n*=3.

Transgenic and WT plants were subjected to drought stress conditions by withholding water at the vegetative stage. Although drought stress resulted in a general decrease of the cwInv activity, transgenic plants showed significantly higher cwInv activity, similar to or even higher than that of the WT plants under control conditions (Fig. 1B). After 7 d of drought stress, *CIN1* plants remained turgid whereas WT plants partially wilted (Fig. 1C, left), and the same effect was observed at the flowering stage 2 d after withholding water (Fig. 1C, right). This strong difference in drought tolerance was even more apparent in seedlings after 15 d of withholding water under uncontrolled conditions in the greenhouse (Fig. 1D, left) and re-watering for 1 d (Fig. 1D, right). Shoot dry weight and leaf area did not differ significantly between WT and transgenic tomato plants growing under drought stress (Fig. 1E, F). Measurements of the root zone water potential (Fig. 2A) revealed that the WT and the *CIN1-*8 line dried the substrate faster than the other *CIN1* lines. Significant differences were observed from day 5 without watering onwards until the end of the drought experiment. The *CIN1-*8 line behaved as WT plants in all subsequent experiments and served as an aphenotypic transgenic control line. Unlike other abiotic stresses, water availability is directly related to productivity through the maintenance of healthy leaves. Although the RWC was reduced during the drought period (Fig. 2B), transgenic plants were superior to WT plants in maintaining soil water potential and consequently leaf RWC, even though they partially wilted.

To gain additional insights into the effect of cwInv in plants expressing *pInvLp6g::CIN1*, accumulated whole-plant transpiration, WUE, stomatal conductance (*g*_s_), chlorophyll fluorescence (*F*_v_/*F*_m_), and photosynthetic rate (*A*) were measured and compared. Accumulated transpiration measured during the drought period was significantly reduced in most of the transgenic lines (by 30%) with respect to the WT (Fig. 2C). Plants expressing *pInvLp6g::CIN1* exhibited a significantly increased WUE (30–50%), measured as the ratio of biomass produced to water used (Fig. 2D). Interestingly, under control conditions, *g*_s_ was significantly lower in the transgenic plants (up to 50% in line *CIN1-*93) compared with the WT plants. In contrast, at the end of the stress period, *g*_s_ was significantly higher in the *CIN1* plants, due to increased water content in the substrate (Fig. 2E).

**Fig. 3.**
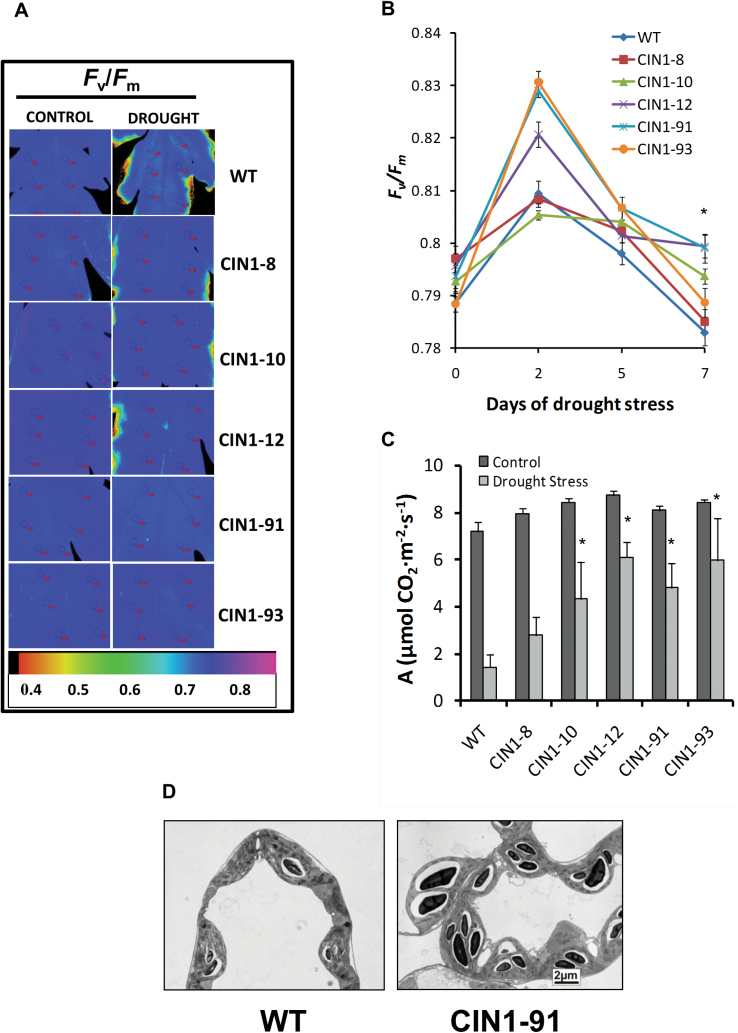
Chlorophyll fluorescence imaging indicating the maximum quantum yield (*F*_v_ */F*_m_) of photosystem II in leaves of WT and *CIN1* plants under normal watering regimes and after 9 d of drought stress (A), and evolution of leaf *F*_v_ */F*_m_ during the drought period (B). Photosynthetic rate in leaves of WT and *CIN1* plants under normal watering regimes and after 9 d of drought stress (C). Transmission electron microscopy images of tomato leaves subjected to 9 d of drought stress showing starch formation (D). Data are presented as means ±SE, *P<0.05, one-way ANOVA, *n*=3.

Furthermore, regarding photosynthetic parameters, analyses of the spatio-temporal changes in (Fig. 3A) and absolute values of (Fig. 3B) *F*_v_ */F*_m_ revealed that chlorophyll fluorescence was higher in the *CIN1* plants than in the WT throughout the drought period. In agreement with this, the photosynthetic rate (*A*) was less reduced in *CIN1* plants (30%) than in the WT plants (75%) and the aphenotypic line *CIN1-*8 (60%) (Fig. 3C). Interestingly, ultrastructural analysis by transmission electron microscopy revealed an increase in sugar storage since *CIN1-*91 leaves presented distinct and larger starch grains than the WT under drought stress (Fig. 3D).

### *CIN1* expression affects sugar and antioxidant metabolism

**Fig. 4.**
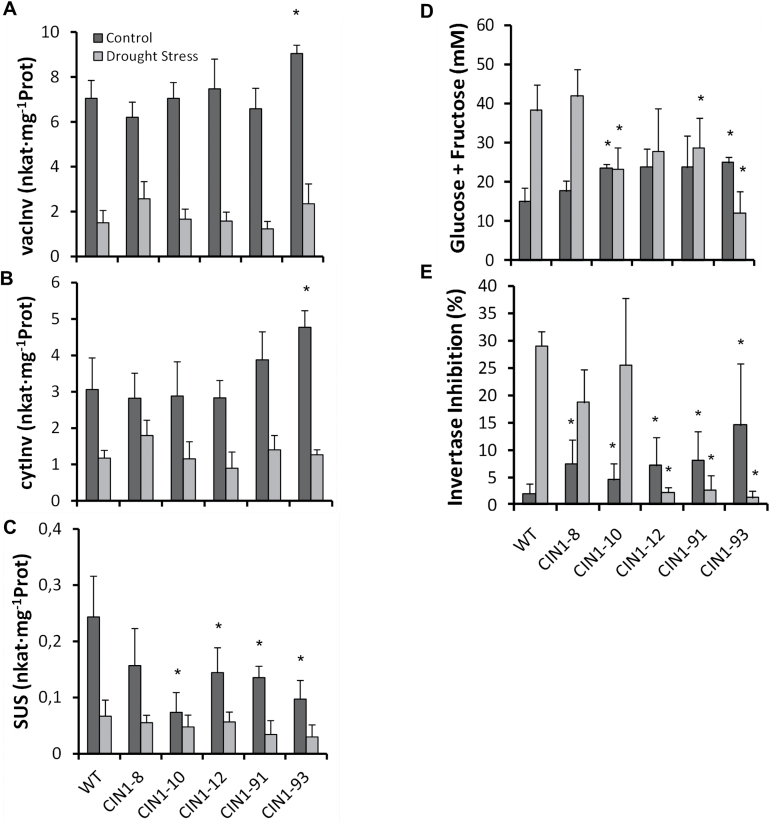
Vacuolar invertase (A), cytoplasmic invertase (B), and sucrose synthase (C) activities, hexose (glucose+fructose) concentrations (D), and invertase inhibitor activity (E) in mature leaves of WT and *CIN1* plants under normal watering regimes and after 9 d of drought stress. Data are presented as means ±SE, *P<0.05, one-way ANOVA, *n*=3.

**Fig. 5.**
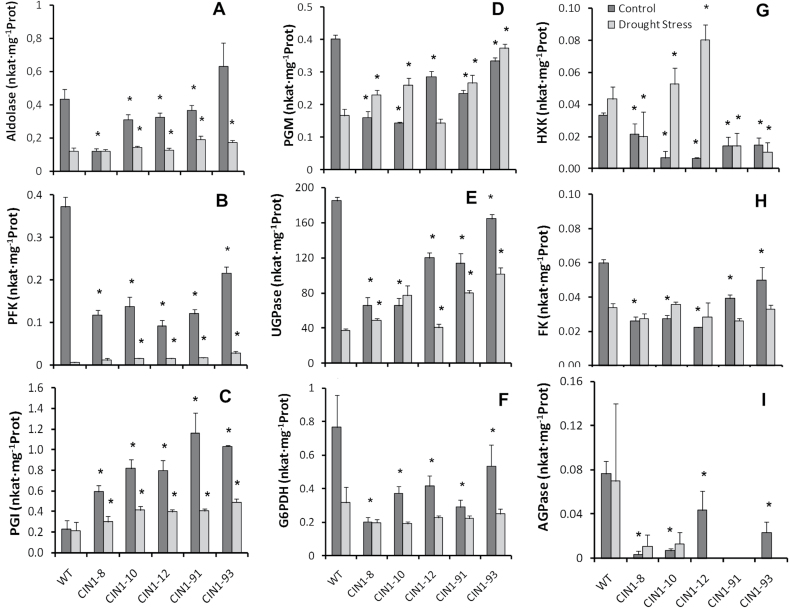
Aldolase (A), phosphofructokinase (B), phosphoglucoisomerase (C), phosphoglucomutase (D), UDP-glucose pyrophosphorylase (E), glucose-6-phosphate dehydrogenase (F), hexokinase (G), fructokinase (H), and ADP-glucose pyrophosphorylase (I) activities in mature leaves of WT and *CIN1* plants under normal watering regimes and after 9 days of drought stress. Data are presented as means ±SE, *P<0.05, one-way ANOVA, *n*=3.

In contrast to cwInv (Fig. 1B), the activity of the other two invertase isoenzymes, vacInv and cytInv, was not or only weakly affected (Fig. 4A, B); the sucrose synthase activity was significantly lower in the transgenic plants under control conditions (Fig. 4C). Despite the increased cwInv activity in the *CIN1* plants, fructose and glucose contents in the leaf were lower than those of the WT and the aphenotypic line *CIN1-*8 under drought stress conditions (Fig. 4D). Invertase inhibitor activity was significantly higher in *CIN1* plants than in the WT under normal watering regimes, especially in line *CIN1-*93 with the highest cwInv activity (Fig. 4E). Under drought stress, a significant increase of the invertase inhibitor activity was observed in the WT and, to a lower extent, in the *CIN1-*8 and *CIN1-*10 lines, while in the other *CIN1* lines, a decrease was detected (Fig. 4E). To determine the effect of the transgene expression on metabolism, in addition to the sucrolytic activities, a set of nine additional key enzymes of primary carbohydrate metabolism was tested (Fig. 5). In general, these enzyme activities were lower under drought conditions. With the exception of phosphoglucoisomerase, all other activities were reduced in the transgenic plants under control conditions compared with the WT, although to a different extent. Under drought, the transgenic plants were characterized by higher activities of the glycolytic enzymes aldolase, phosphofructokinase, phosphoglucoisomerase, phosphoglucomutase, and UDP-glucose-pyrophosphorylase (Fig. 5A–E). The activity of glucose-6-phosphate dehydrogenase was lower in the *CIN1* plants under both control and drought stress conditions (Fig. 5F). Hexokinase, fructokinase, and ADP-glucose-pyrophosphorylase activities did not show consistent changes under water stress conditions (Fig. 5G–I).

**Fig. 6.**
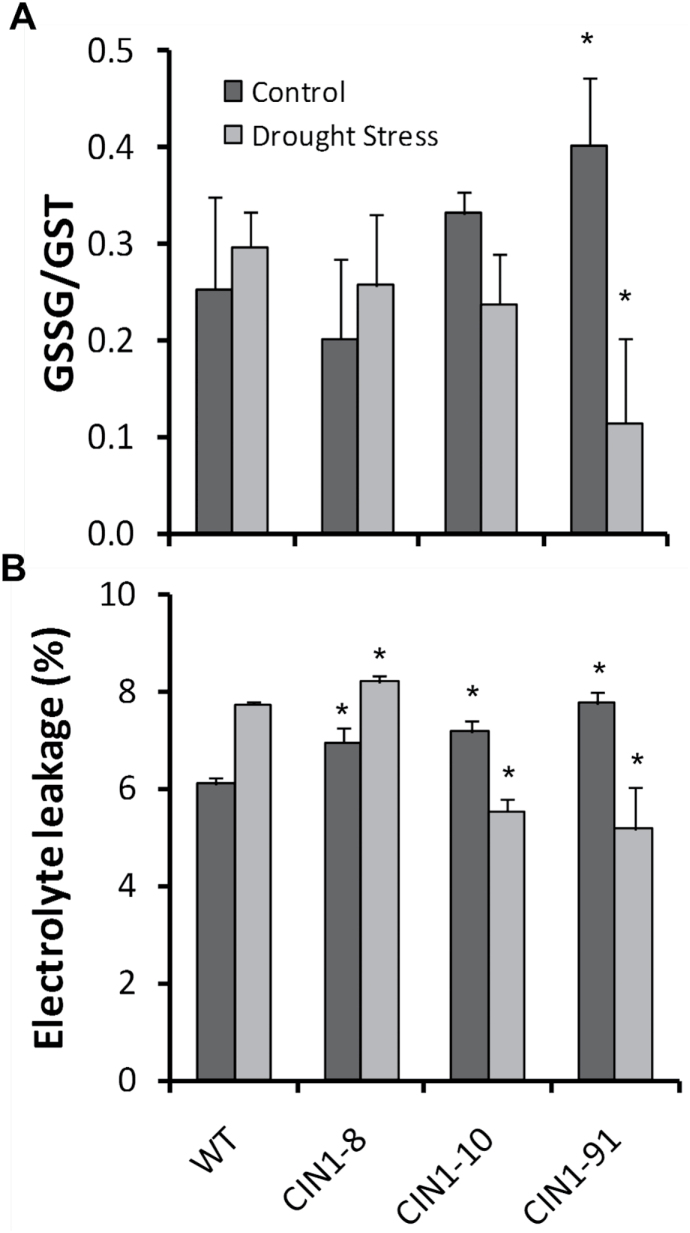
Ratio of oxidized to total glutathione (A) and electrolyte leakage (B) in mature leaves of WT and *CIN1* plants under normal watering regimes, and after 9 d of drought stress. Data are presented as means ±SE, *P<0.05, one-way ANOVA, *n*=3.

**Fig. 7.**
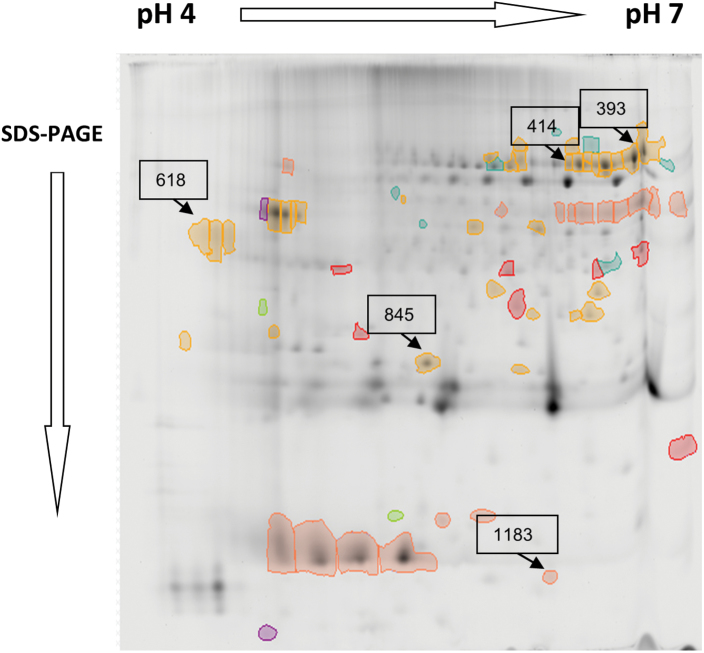
Reference 2D electrophoretic pattern of mature leaf apoplast from WT plants and the *CIN1-*91 line growing under normal watering regimes and after 9 d of drought stress. Proteins were resolved using a linear pH gradient of 4–7 in the first dimension and 12% SDS–PAGE in the second dimension. Selected spots are indicated with arrows.

During stress, disruption of cellular homeostasis is accompanied by the generation of reactive oxygen species (ROS), and the extent of stress-induced damage can be attenuated by the action of the cell’s antioxidant systems, including glutathione and enzymes capable of scavenging ROS. The oxidized state of glutathione increased during drought in the WT and the aphenotypic line *CIN1-*8, but decreased in the other transgenic *CIN1* lines (Fig. 6A). Apoplastic POX and SOD activities were strongly reduced by drought stress in the WT but were maintained or induced in *CIN1* plants (Table 1). This was related to the performance of the different *CIN1* lines and the electrolyte leakage (membrane stability) during the drought period. In this sense, cell membrane damage under drought stress was more severe in the WT and the aphenotypic *CIN1-*8 line than in the other *CIN1* lines analysed (Fig. 6B). Proteomic analysis (Fig. 7; Table 2) confirmed a high expression for one POX isoform (spot 618) in *CIN1* plants and other proteins related to plant stress defence responses: chitinase (spot 845) and proteases of the subtilisin-like clan (spots 393 and 414).

**Table 1.** Superoxide dismutase (SOD) and peroxidase (POX) activities (U g^–1^ FW), and total peroxidase activity (expressed as a percentage of the control) in tomato leaves of WT and CIN1 plants under normal and drought stress conditions

Genotype	Apoplast		Simplast		Total POX activity
	SOD	POX	SOD	POX	
Control					
WT	2.5±0.1	3.4±0.1	291.9±1.3	51.08±0.4	
*CIN1*-8	1.7±0.0*	2.36±0.2*	245.3±23.9	31.03±0.7*	
*CIN1*-10	1.36±0.1*	2.23±0.4*	201.5±6.4*	19.78±1.1*	
*CIN1-*91	2.41±0.0	3.26±0.0	210.1±7.9*	28.29±0.6*	
Drought stress					
WT	1.48±0.2 (58)	2.09±0.1 (61)	232.5±4.5 (80)	28.96±2.8 (57)	57
*CIN1*-8	1.59±0.1* (94)	4.02±0.1 (170)	263.5±0.0* (107)	27.62±1.0 (89)	95
*CIN1*-10	1.74±0.1* (128)	2.61±0.2* (117)	149.4±0.2* (74)	22.3±1.2* (113)	113
*CIN1*-91	2.11±0.0* (85)	1.6±0.1* (49)	234.1±3.1 (111)	30.2±1.0 (107)	101

Data are presented as means ±SE, *P<0.05, one-way ANOVA, *n*=3.

**Table 2.** Apoplastic protein identification from the proteomic analysis in leaves of the WT and the *CIN1*-91 line under control and drought stress conditions

Spot	Accession no.	Protein name	Spot normalized volumes			
			Control		Drought stress	
			WT	*CIN1*-91	WT	*CIN1*-91
618	6723685	Peroxidase	0.54	1.33	1.24	1.52
845	31088232	Chitinase	0.52	1.05	0.82	1.91
393	2230959	Subtilisin-like protease	0.41	1.26	0.77	1.86
414	219760217	Subtilisin-like protease	0.31	0.84	0.70	1.19
1183	461978	Glucan endo-1,3-β-glucosidase A	1.99	1.05	1.17	0.64

### Changes in leaf hormonal balance

**Fig. 8.**
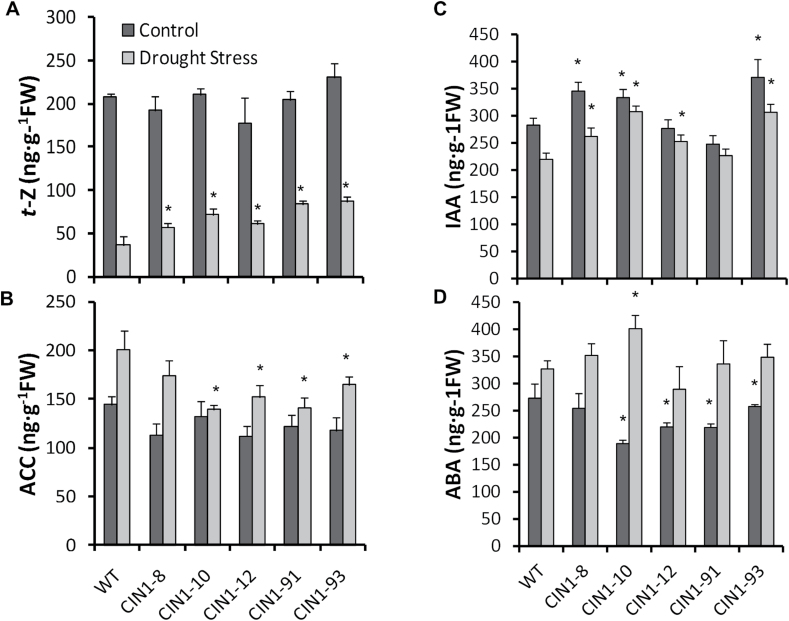
*trans*-Zeatin (t-Z; A), the ethylene precursor 1-aminocyclopropane-1-carboxylic acid (ACC; B), indoleacetic acid (IAA; C), and abscisic acid (ABA; D) concentrations in tomato leaves of WT and *CIN1* plants under normal watering regimes and after 9 d of drought stress. Data are presented as means ±SE, *P<0.05, one-way ANOVA, *n*=3.

Leaf concentrations of the active cytokinin (CK) *trans*-zeatin (*t*Z) and the ethylene precursor 1-aminocyclopropane-1-carboxylic acid (ACC) were analysed in control and water-stressed plants at the end of the experiment. Although *t*Z levels were reduced under drought stress, *CIN1* lines that showed a strong phenotype also presented a significant increase in *t*Z compared with WT plants (Fig. 8A). Drought stress resulted in a general increase in the ACC concentrations in WT and *CIN1* plants, but remained significantly lower (by 25%) in the *CIN1* lines, except for the aphenotypic *CIN1-*8 line (Fig. 8B). While auxin levels were slightly increased in the transgenic plants (Fig. 8C), no clear trend was evident for the abscisic acid (ABA) levels (Fig. 8D).

### *CIN1* expression modifies the metabolite profile under drought stress

**Fig. 9.**
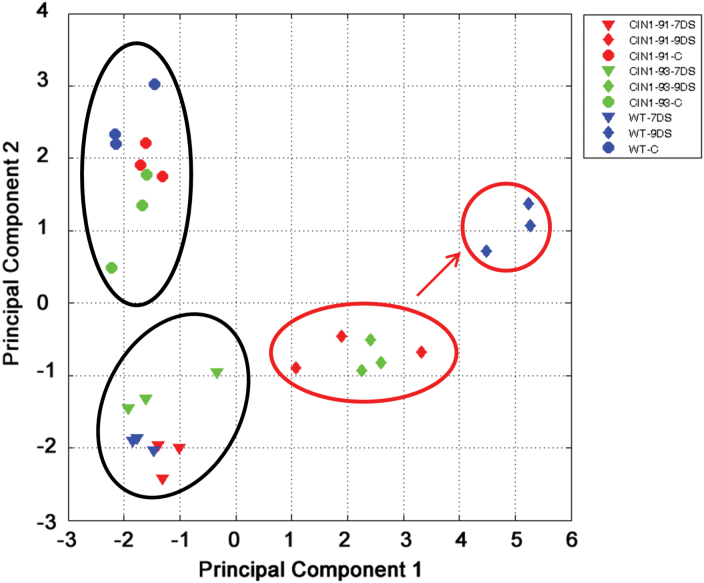
Principal component analysis of those metabolites analysed in mature leaves of the WT and two *CIN1* lines (*CIN1-*91 and *CIN1-*93) which showed significant differences between control (C) and drought stress (DS) conditions. Circles indicate samples that cluster together. The arrow indicates a shift of the WT compared with *CIN1* plants following 9 d of drought stress.

To characterize further the mechanism of drought stress tolerance in *CIN1* plants, metabolite profiling was performed. This identified 100 compounds, of which 22 showed significant changes due to drought stress conditions (Table 3). *CIN1* plants showed specific accumulation of phosphorylated sugar intermediates glucose-6-phosphate and fructose-6-phosphate, organic acids, and phenolic compounds. Five out of six mono- and disaccharides and a sugar-alcohol were reduced in the leaves of transgenic lines under drought conditions, whereas amino acids showed a differential response (Table 3). This set of metabolites was subjected to principal component analysis (PCA). Although the *CIN1* plants clustered together with the WT plants under control conditions, at the end of the drought period *CIN1* plants clearly clustered separately from WT plants (Fig. 9). The fact that the stressed *CIN1* plants clustered in between plants under control conditions and stressed WT plants for the principal component 1 (PC1) indicates that *CIN1* plants are less affected by drought stress conditions (Fig. 8).

**Table 3.** Ratio between leaf metabolite concentration after 9 d of drought stress (DS) and control conditions (C) of selected metabolites that show significant changes

	Metabolites	Ratio (DS/C)		
		WT	*CIN1*-91	*CIN1*-93
Carbohydrates and derivatives	Fructose	14.24	7.05*	8.13*
	Glucose	57.34	18.10*	19.01*
	Sucrose	2.36	1.72*	2.01*
	Galactose	1.78	1.30*	1.00*
	Sorbose	1.04	2.53*	1.39*
	Altrose	2.59	2.18*	1.67*
	Fructose-6-phosphate	0.49	1.83*	1.38*
	Glucose-6-phosphate	0.48	1.76*	1.30*
	Ribitol	2.33	2.08*	1.56*
Organic acids and derivatives	Citric acid	0.31	0.77*	0.83*
	Isoascorbic acid	13.46	18.21*	4.80*
	Threonic acid	1.28	2.18*	1.69*
	Galactonic acid	1.53	4.81*	3.45*
Shikimate and phenolics	Shikimic acid	0.37	1.44*	1.59*
	3-*trans*-caffeoylquinic acid	1.61	3.21*	3.65*
	5-*trans*-caffeoylquinic acid	0.90	1.65*	3.16*
	Quinic acid	1.02	3.75*	3.78*
Amino acids	Asparagine	0.67	1.75*	0.96*
	Aspartic acid	0.65	1.63*	1.04*
	Tryptophan	2.66	1.46*	0.82*
	Leucine	2.05	0.77*	0.86*
	Tryrosine	2.38	1.47*	1.16*

Data are presented as means, *P<0.05, one-way ANOVA, *n*=3.

## Discussion

Although plant responses to drought stress have been intensively studied for many years, progress in the development of tolerant crops has been slow. A number of genes, including those involved in the production of osmotic adjustment, detoxification of ROS, and transcription factors, have been employed to increase drought tolerance in transgenic plants (Munns and Tester, 2008; Faize *et al.*, 2011; Skirycz *et al.*, 2011). However, the success rate is typically very low, probably due to the lack of knowledge about the mechanisms that are controlling growth under water stress conditions. In general, adaptation or engineered tolerance to drought is accompanied by adverse effects on development and yield. Therefore, maintaining growth, photosynthesis, and metabolism are the major goals to minimize the impact of abiotic stress on crop yield. Here it is shown that ectopic expression of the *CIN1* gene encoding a cwInv from *C. rubrum* resulted in various metabolic changes that improved tomato adaptation to drought stress conditions by increasing WUE.

Many studies demonstrated that decreased stomatal conductance and transpiration under drought stress conditions is the main factor limiting photosynthesis (Chernyad’ev, 1997; Chaves *et al.*, 2009; Galmés *et al.*, 2011), and assimilate transport in phloem sets conditions for gas exchange (Nikinmaa *et al.*, 2013). The present results show that *CIN1* plants had limited whole-plant transpiration during drought stress, and importantly WUE increased (Fig. 2). In fact, under control conditions, stomatal conductance was reduced in the *CIN1* plants (Fig. 2E), leading to reduced water consumption during the drought period, while photosynthetic activity was maintained (Fig. 3A–C). In contrast, the increased stomatal conductance observed in the transgenic plants at the end of the drought period is explained by the higher water availability in the soil, compared with the WT plants. Therefore, it seems that *CIN1* plants present a better control of stomatal closure explained by metabolic factors since it has been demonstrated that sugar levels and sucrolytic activities play important roles in guard cell function with impacts on WUE (Antunes *et al.*, 2012). Indeed, it has been reported that vacuolar invertase activity was higher in guard cells than in other epidermal cells and it plays an important role in regulating stomatal aperture in *Arabidopsis* (Ni, 2012). Therefore, the increased cwInv activity (Fig. 1B) and hexose concentrations (Fig. 4D) observed in the leaves of *CIN1* plants under normal watering regimes could provoke a reduction in the stomatal conductance retaining water for the drought period. Similarly, it has been reported that overexpression of the *Arabidopsis* trehalase *AtTRE1* gene, the only enzyme known in this species specifically to hydrolyse trehalose into glucose, leads to increased drought stress tolerance through an induction of stomatal closure (Van Houtte *et al.*, 2013). Other trehalose enzymes and intermediates, such as trehalose-6-phosphate, have also been linked to abiotic stress tolerance in tomato (Delorge *et al.*, 2014). In contrast, the lower stomatal conductance observed in the WT plants at the end of the drought period can be explained by the higher level of stress due to reduced soil moisture compared with *CIN1* plants (Fig. 2A).

After the initial assimilate accumulation under stress, photosynthesis may be inhibited by metabolic factors, stomatal-derived signals, and other regulatory mechanisms, while stress-induced leaf senescence limits whole-plant photosynthesis (Munns and Tester, 2008), as seems to be the case in the WT and the aphenotypic *CIN1-*8 line (Fig. 3A–C) due to the expected hexose accumulation (Fig. 4D). Avoiding feedback inhibition of photosynthesis by co-ordinating the assimilate transport between source and sink tissues by invertases and/or partitioning of sugars towards starch accumulation may maintain photosynthetic activity under harmful environmental conditions (Stitt, 1991; Koch, 1996) and thus delay leaf senescence. Recently, it has been shown that engineered drought tolerance in tomato is reflected in chlorophyll fluorescence emission signatures (Mishra *et al.*, 2012), and similar changes were also observed in the *CIN1* plants (Fig. 3A, B). An increase in the concentrations of the invertase products fructose and glucose would be expected, as has been recently shown in rice plants that overexpress the cwInv gen *GIF1* related to induced pathogen defence (Sun *et al.*, 2014). Strikingly, despite higher cwInv activities (Fig. 1B), fructose and glucose contents in droughted leaves of *CIN1* plants were lower than those in the WT, but similar to those of control leaves (Fig. 4D). These results resemble the effect of ectopic expression of cwInv in delaying natural or light-induced senescence in transgenic tobacco plants (Balibrea *et al.*, 2004). The unexpected finding that an increase in cwInv in *CIN1* plants does not result in an increase in hexose steady-state concentrations would explain the maintenance of photosynthetic activity despite the activation of sink metabolism. Therefore, the delay of senescence induced by cwInv in *CIN1* plants may be related to an activation of the metabolic carbohydrate flux. The resulting higher rate of sugar utilization causes a decrease of hexose levels in the transgenic plants (Balibrea *et al.*, 2004). Thus, despite the activation of sink metabolism, it seems that the hexose concentration does not reach the threshold level that would result in the feedback inhibition of photosynthetic gene expression. This provides a mechanism to uncouple the usually observed inverse and co-ordinated regulation of source and sink metabolism. The apparent stimulation of carbohydrate fluxes is also reflected by the finding that the transgenic plants were able to maintain higher activities of additional key enzymes of primary carbohydrate metabolism under drought stress conditions, such as aldolase, phosphoglucomutase, phosphofructokinase, phosphoglucoisomerase, and UDP-glucose pyrophosphorylase (Fig. 5). Those enzymes are involved in sucrose and starch biosynthesis from triose-phosphate resulting from the higher photosynthetic activity, thus increasing the source activity. For example, it has been demonstrated that aldolase, a key enzyme for energy production, plays important roles in the tolerance to drought and salt stress by activating glycolysis (Fan *et al.*, 2009). Thus, *CIN1* plants were able to maintain metabolic fluxes of primary carbohydrate metabolism in support of growth and energy production for stress tolerance compared with WT plants, also reflected by higher levels of the phosphorylated intermediates fructose-6-phosphate and glucose-6-phosphate (Table 3; Fig. 9). Evidence for carbon flux shortage under water stress in sink organs (nodules) has been shown in pea plants (Gálvez *et al.*, 2005). Apparently, drought tolerance is related to a fine-tuned interaction and balance between extracellular hydrolysis by the cwInv and the metabolic flux of the sink cell to avoid the accumulation of carbohydrates under harmful conditions. Furthermore, the large starch grains in leaves from *CIN1* plants (Fig. 3D) indicate that changing the cycle of starch synthesis and breakdown and thus metabolic channelling could be an additional mechanism to contribute to drought stress tolerance in *CIN1* plants (Sulpice *et al.*, 2009) in order to maximize carbon uptake and growth, while minimizing osmotic impacts on photo-inhibition and photo-oxidation.

Photo-oxidative damage and premature leaf senescence are common effects of abiotic stress challenges, inhibiting growth (Munns, 2002). Photo-inhibition and photo-oxidation are caused by the impaired consumption of NADPH by the Calvin cycle, with the subsequent transfer of photosynthetic electrons from over-reduced ferredoxin to oxygen (Mehler reaction) producing ROS that damage cell structures (Stitt, 1991; Paul and Foyer, 2001). SOD and POX activities were maintained in *CIN1* plants under drought stress (Table 1), especially apoplastic SOD, which agrees with the reported accumulation of superoxide radicals under salt stress conditions in this compartment (Hernández *et al.*, 2001). It has been reported that glucose and CK agonistically regulate POX activity in *Arabidopsis* (Kushwah and Laxmi, 2014), thus explaining the higher and stable levels of this protein in the *CIN1* plants (Table 2). Furthermore, many reports indicate that the ratio between reduced and oxidized glutathione (GSH/GSSG) is an effective marker of cellular redox homeostasis and may be involved in ROS activity perception by plants under drought conditions (Labudda and Azam, 2014). In this way, GSH/GSSG play a direct or indirect key role in regulating and signalling at the transcriptional and/or post-translational level due to the interaction of these molecules with other cellular redox systems such as the aforementioned enzymes SOD and POX (Anjum *et al.*, 2012), as well as with the hormonal balance (Miao *et al.*, 2006). Maintaining the activity of these two ROS-scavenging enzymes, together with decreased concentrations of oxidized glutathione (Fig. 6A) and reduced electrolyte leakage (Fig. 6B), resulted in a better control of ROS levels and sustained membrane protection in drought-stressed *CIN1* plants, such as reported in the senescence-regulated CK-overproducing (*SARK2::IPT*) tobacco plants in response to drought (Rivero *et al.*, 2007). A possible link between antioxidants and carbohydrate metabolism could be the glucose-6-phosphate dehydrogenase, which is essential for maintaining the cellular redox balance and shown to be critically involved in salt and drought stress responses (Dal Santo *et al.*, 2012). Possibly due to the post-translational regulatory mechanism involved in the activation of this enzyme, the measured activities do not reflect the *in vivo* situation.

Sugar and hormones such as CKs are fundamental to plants and regulate a number of similar processes agonistically (Kushwah and Laxmi, 2014). It has been recently reported that glucose induces CK biosynthetic (*IPT3*) and perception (*AHK4*) genes, while it represses some CK-degrading enzymes (*CKX5*) in *Arabidopsis* (Kushwah and Laxmi, 2014). Ectopic expression of *CIN1* under the control of the senescence-associated promoter *SAG12* increased source strength and delayed developmentally regulated leaf senescence in tobacco, and identified cwInv as an essential component of the CK-mediated delay of senescence (Balibrea *et al.*, 2004). More recently, the extreme drought tolerance observed in tobacco plants overexpressing the CK biosynthesis gene *IPT* was also related to delayed senescence due to increased CK levels, without affecting ABA levels (Rivero *et al.*, 2007), as it occurred in the *CIN1* plants (Fig. 8D). Furthermore, rice plants overexpressing the *IPT* gene showed increased water stress tolerance and grain yield due to CK-mediated source/sink modifications (Peleg *et al.*, 2011). Therefore, delayed leaf senescence of *CIN1* plants under drought stress could be explained by both higher cwInv activity (Fig. 1B) and higher *t*Z levels in *CIN1* leaves (Fig. 8A), thus mimicking the *IPT*-overexpressing plants. Additionally, stressed *CIN1* plants showed a significant decrease in the ethylene precursor ACC (Fig. 8B). Ethylene has been long considered a stress-related hormone that mediates leaf senescence under abiotic stress conditions (Munné-Bosch and Alegre, 2004), probably by inhibiting cwInv (Ghanem *et al.*, 2008). Because cwInv appears to activate metabolic carbohydrate fluxes (Balibrea *et al.*, 2004) that repress ethylene biosynthesis (Mayak and Borochov, 1984), probably through glucose suppression of ACC oxidase activity (Hong *et al.*, 2004), leaf senescence could additionally be delayed by the reduction of ethylene levels. Together these data indicate that the physiological changes conferred by *CIN1* overexpression during the vegetative stage are closely connected with hormonal (CKs and ethylene, but not ABA) and sugar metabolism, suggesting that a causative effect of cwInv on the regulation of plant hormonal balance and function cannot be ruled out, as has been recently demonstrated in fruits of tomato plants subjected to salt stress (Albacete *et al.*, 2014*a*).

Post-translational relief of invertase from inhibition by a proteinacous inhibitor has been implicated in abiotic stress tolerance (Ruan *et al.*, 2010). The *in vivo* functionality and the physiological significance of these inhibitors in plant growth, development and stress responses have been demonstrated only recently (Jin *et al.*, 2009; Bonfig *et al.*, 2010). Such post-translational regulation is particularly relevant to cwInv because these proteins are intrinsically stable due to their glycosylation (Rausch and Greiner, 2004). Increased invertase inhibitor activity in the *CIN1* plants under control conditions probably reflects feedback regulation to decrease the ectopic transgenic cwInv activities and minimize negative impacts on phloem loading (Fig. 4E). In contrast, drought stress resulted in a strong decrease of the invertase inhibition, thus linking the local induction and/or maintenance of the sink strength to stress responses by derepression of the invertase activity present.

In summary, the results show that although the promoter employed should confer predominantly expression in developing tomato fruits (Albacete *et al.*, 2014*a*), a weak vegetative *CIN1* expression was already sufficient to confer tolerance towards drought stress without affecting plant fitness under optimal growth conditions. A reduced water use and an increased source activity explained by changes in metabolic fluxes, and hormonal and redox status suggest that the physiological mechanisms regulated by cell wall invertase play a key role in abiotic stress adaptation. The results presented in this study are very promising because enhanced WUE and plant adaptation to drought stress would contribute to the development of new varieties with reduced yield penalties in the most economically important horticultural areas, where water resources are scarce.
