# Ectopic overexpression of the cell wall invertase gene *CIN1* leads to dehydration avoidance in tomato

**DOI:** 10.1093/jxb/erv134

**Published:** 2015-05-21

**Authors:** Alfonso Albacete, Elena Cantero-Navarro, Dominik K. Großkinsky, Cintia L. Arias, María Encarnación Balibrea, Roque Bru, Lena Fragner, Michel E. Ghanem, María de la Cruz González, Jose A. Hernández, Cristina Martínez-Andújar, Eric van der Graaff, Wolfram Weckwerth, Günther Zellnig, Francisco Pérez-Alfocea, Thomas Roitsch

**Affiliations:** ^1^ Department of Plant Nutrition, CEBAS-CSIC, Campus de Espinardo, 30100 Murcia, Spain; ^2^ Institute of Plant Sciences, Department of Plant Physiology, University of Graz, 8010 Graz, Austria; ^3^ Department of Plant and Environmental Sciences, Copenhagen Plant Science Centre, University of Copenhagen, Højbakkegård Allé 13, DK-2630 Taastrup, Denmark; ^4^ Centro de Estudios Fotosintéticos y Bioquímicos, Universidad Nacional de Rosario, Suipacha 531, 2000 Rosario, Argentina; ^5^ Departamento de Agroquímica y Bioquímica, Facultad de Ciencias, Universidad de Alicante, 03080 Alicante, Spain; ^6^ Department of Molecular Systems Biology, Faculty of Life Sciences, University of Vienna, 1090 Vienna, Austria; ^7^ Instituto de Bioquímica Vegetal y Fotosíntesis, Universidad de Sevilla, CSIC, 41092 Sevilla, Spain; ^8^ Department of Fruit Breeding, CEBAS-CSIC, Campus de Espinardo, 30100 Murcia, Spain; ^9^ Global Change Research Centre, Czech Globe AS CR, v.v.i.., Drásov 470, Cz-664 24 Drásov, Czech Republic

*Journal of Experimental Botany*, Vol. 66, No. 3 pp. 863–878, 2015, doi: 10.1093/jxb/eru448

In figure 2 of the above paper, the Y-axis labels of the graphics D and E were incorrectly typed:

- Graphic 2D: water use efficiency (WUE) was calculated on the basis of fresh weight and, therefore, the correct Y-axis label is ‘WUE (gFW·ml^–1^ TW)’
- Graphic 2E: the units of stomatal conductance were incorrectly typed; the correct Y-axis label is ‘g_s_ (mol H_2_O·m^–2^·s^–1^)’

The corrected figure 2 is reprinted here:



